# WASH and NTDs: Outcomes and lessons learned from the implementation of a formative research study in NTD skin co-endemic communities in Benin

**DOI:** 10.3389/fmed.2023.1022314

**Published:** 2023-02-28

**Authors:** Zoulkifl Salou Bachirou, Zinsou Franck Mignanwande, Hervé Bokossa, Horace Degnonvi, Parfait Djossou, Flora Hondjrebo, Hermione Amoukpo, Esai Gimatal Anagonou, Inès Agbo, Damien Toffa, Rafiatou Ba, Anna Gine, Gabriel Diez, Roch Christian Johnson

**Affiliations:** ^1^Interfaculty Center for Training and Research in Environment for Sustainable Development (CIFRED), University of Abomey-Calavi, Abomey Calavi, Benin; ^2^Anesvad Foundation, Bilbao, Spain

**Keywords:** WASH, skin NTD, STH, wound care, formative research, Benin

## Abstract

Neglected Tropical Diseases (NTDs) are a diverse group of bacterial, viral, parasitic and fungal diseases affecting people, most of whom live below the poverty threshold. Several control strategies are defined against these diseases, including chemotherapy and Water, Hygiene and Sanitation (WASH). This study assesses the effect of promoting hygiene and sanitation on soil-transmitted helminthiasis s and NTDs of the skin. It took place in the communes of Ze, Lalo, and Zangnanado, three municipalities located in the south of Benin. This is a formative research that took place in three phases. The first phase entailed a baseline informations and situational analysis of the state of hygiene and health, using soil-transmitted helminthiasis and wound hygiene practices as cases studies. In the second phase, interventions to promote improved hygiene and sanitation were implemented. The third phase was devoted to post-intervention evaluation. The situation analysis showed that the prevalence of soil-transmitted helminthiasis was 6.43 and 7.10% in the municipalities of Ze and Lalo, respectively. In the communes of Zangnanado, the most common wound management practices identified were: putting sand or ashes in the wounds to keep flies away, the use of medicinal plants and the application of powder from antibiotic capsules for wound dressing. The post-intervention evaluation showed a decrease in the prevalence of soil-transmitted helminthiasis from 6.43 to 1.19% in the municipality of Lalo and from 7.10 to 1.75% in the municipality of Ze. In the commune of Zangnanado, a significant shift in wound management practices was noted, which led to the healing of several chronic wounds. This research supports the evidence that WASH-based interventions are very important to tackle neglected tropical diseases NTDs in addition to specific diseases based interventions.

## Introduction

Neglected Tropical Diseases (NTDs) affect 2.7 billion people, most of whom live below the poverty line (1). Although particular interest has been given to these diseases since the 2000s, given the low-cost interventions available (2), they persist. Indeed, NTDs remain present in disadvantaged communities, where they maintain populations in a vicious circle of intergenerational transmission of poverty (3).

Currently, WHO focuses on twenty (20) NTDs or groups of endemic diseases, including leprosy, Buruli ulcer, soil-transmitted helminthiases (STH), envenomations (4). STH infections are a group of intestinal worms consisting of *Ascaris lumbricoides*, *Trichuris trichiura*, *Necator americanus*, *Ancylostoma duodenale*, and *Ancylostoma ceylanicum* (5). These species are among the most frequent infections in populations, affecting more than 1.5 billion individuals worldwide (6). Infection occurs through accidental ingestion of eggs of *Ascaris lumbricoides*, and *Trichuris trichiura* and occasionally *Ankylostoma duodenale* or penetration into the skin of hookworm larvae present in contaminated soils (7).

The cycle of transmission of NTDs cannot be broken by treatment alone and improving access to Water; Hygiene and Sanitation (WASH) is also important for the control and elimination of these diseases (8, 9). Despite the recognized importance of water and improved sanitation in the NTDs control, surveillance programs for several NTDs have been largely limited to mass drug administration; NTD control interventions through improved WASH are uncommon (10, 11). Whereas, WHO recommends five core strategic interventions to address the burden of NTDs, namely chemoprevention, individual case management, vector control, WASH, veterinary public health (12), it should be noted that 1.6 billion people still do not have access to drinking water at home, 2.8 billion do not have access to safe sanitation facilities and 1.9 billion do not have access to basic handwashing facilities at home (13). Benin, a West Africa country, is endemic to some NTDs. Thus, among the fourteen neglected tropical diseases (NTDs) of the WHO region of Africa, those which are endemic in Benin are onchocerciasis, lymphatic filariasis, schistosomiasis, human African trypanosomiasis, geohelminthiasis, dracunculiasis, leprosy, Buruli ulcer, trachoma; loiasis (14). To fight or control these diseases, chemotherapy and individual case management are the strategies used until now. The cross-cutting approach of improving access to WASH and education for behavior change have hardly been tested to combat these NTDs; whereas, according to the WHO, close epidemiological associations exist between insufficient access to water, sanitation and hygiene and a wide range of conditions, including NTDs (15). The objective of this study is to investigate the effect of hygiene and sanitation promotion interventions on soil-transmitted helminth infections and skin-manifesting NTDs.

## Materials and methods

### Study design

**FIGURE 1:**
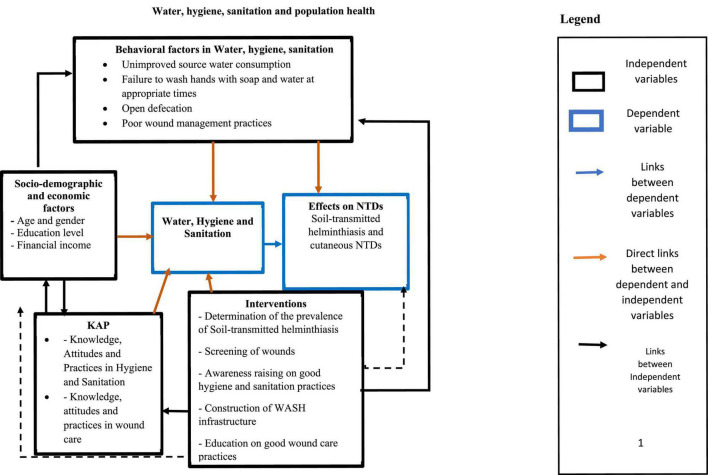
Conceptual framework of the study.

We conducted a formative research study in three phases. The first phase consisted of collecting baseline information related to the prevalence of soil-transmitted helminthiasis in the municipalities of Lalo and Ze and on wound hygiene practices related to the management of NTDs with cutaneous manifestations (leprosy, Buruli ulcer) in the municipality of Zagnanado. In the second phase, interventions to promote access to WASH and wound hygiene were implemented. A post-intervention evaluation was finally carried out. Figure 1 represents the conceptual framework of the study.

### Setting and study population

The study took place in the municipalities of Lalo, Ze and Zagnanado, respectively in the departments of Couffo, Atlantique, and Zou in southern Benin. In the municipalities of Ze and Lalo, the study population targeted pupils from seven (07) Public Primary Schools. These schools are Ahomadégbé/A, Ahomadégbé/B, Adjaigbonou, in the municipality of Lalo; then schools Sédjè-Dénou/A, Sédjè-Dénou/B, Aguiakpa, and Agbohounsou, in the municipality of Ze. To be included in the study, students must be present at school on the day of data collection and then give their consent and that of their parents. In the municipality of Zagnanado, the general population and particularly people exposed to NTDs with cutaneous manifestations are the target of the study. People must have given their consent to participate in the study before being included.

### Sampling and data collection

A sample of two hundred and forty-nine (249) pupils from Lalo schools and three hundred and fifty-two (352) from Ze schools was enrolled based on the inclusion criteria mentioned above. For the study on the management of NTDs with cutaneous manifestations in the municipality of Zagnanado, 1158 subjects were included.

### Variables and measurement

For the study on the prevalence of soil-transmitted helminthiasis in the municipalities of Lalo and Ze a questionnaire was used for pupils, then their stools were collected for laboratory analysis. The questionnaire made it possible to collect information related to the socio-demographic characteristics and behavior of schoolchildren in terms of hygiene and sanitation. The eggs or parasites were sought in the stools of the schoolchildren using Kato Katz method. The genus and species are then specified in case of presence and the number of eggs and parasites were counted.

Regarding the study on the management of NTDs with cutaneous manifestations in the municipality of Zagnanado, several techniques were used to analyze the practices of home-based wound care practices. These included open and semi-structured interviews, case studies, focus group and then structured observations. Interviews were first conducted with members of the community with wounds, then with members in the community identified as having considerable experience in treating wounds. These techniques have made it possible to make a list of common wound care practices and products used for home-based wound care, including substances commonly used for wound hygiene to clean, treat and dress wounds, as well as to manage sensations of pain, itching, wound-related burns. They also allowed the recognition of danger signs, perceptions of the healing process.

### Implemented interventions

After the baseline informations, several interventions were implemented:

• In schools, improved toilets respecting gender separation, drilling wells for drinking water supply, the awareness of schoolchildren on good hygiene and sanitation practices according to the CHAST approach (Children, Hygiene, and Sanitation Training). A free distribution of albendazole according to WHO standards and recommendations was also offered to all school children, both those who agreed to participate in the study and those who did not give their consent.
• In the municipality of Zagnanado, the interventions implemented focused on raising public awareness of wound screening, home care of wounds, training in wound dressing at home using clean water and soap and provision of dressing kit (soap, shea butter, bandage).

### Data analysis

As far as the investigation of the prevalence of soil-transmitted helminthiasis concerned, the data collected were described by their means and standard deviation when the distribution is normal, median and interquartile range are used if the distribution is asymmetrical. For categorical ones, they are described using proportion. A bivariate analysis was then carried out to seek the existence of association between the dichotomous qualitative dependent variable and the independent variables by adequate parametric tests. The association was found to be significant for independent variables with a *p*-value less than 0.05. In addition, stool samples collected in tubes given to pupils were analyzed in the laboratory using the kato-katz technique (16). This technique was used to determine the prevalence of geohelminthiasis as well as the identification of the different geohelminth species (17, 18).

The reading of the blades was done by two experienced bio-technicians and the quality control carried out by the parasitology department of the National Hospital and University Center (NHUC) of Cotonou. To do this, 10% of the blades collected were randomly drawn and the number of eggs carried by these blades was recounted.

For the study on the management of NTDs with cutaneous manifestations, the qualitative data collected during interviews were fully transcribed. These data were entered in Word software and then compiled. They were then subjected to content analysis.

### Post-interventions assessment

At the end of interventions, we then carried out a post-intervention evaluation of the new prevalence of soil-transmitted helminthiasis in the municipalities of Lalo and Ze according to the same protocol previously described.

A follow-up of the healing of the wounds with the people who benefited from the care and the awareness sessions was also carried out. The main indicators for assessing the effects of interventions were the reduction in the prevalence of soil-transmitted helminthiasis, and behavior changes in the home-based wound healing practices.

## Results

In this section, we presented:

• the results of the baseline study on the prevalence of soil-transmitted helminthiasis and home-based wound hygiene practices;
• interventions implemented;
• the results of the post-intervention assessment.

### Socio-demographic characteristics of respondents in the municipality of Lalo

The socio-demographic characteristics of pupils in the municipality of Lalo are presented in Table 1.

**TABLE 1:** Socio-demographic characteristics of Lalo schoolchildren.

Variables	Modalities	Number (*n* = 249)	Frequency (%)
Schools	Ahomadégbé/A	79	31.7
	Ahomadégbé/B	65	26.1
	Adjaigbonou	105	42.2
Sex	Female	99	39.8
	Male	150	60.2
Age	4–10 years	183	73.5
	11–15 years	66	26.5
School level	CI–CE1	166	66.7
	CE2–CM2	83	33.3
Father’s profession	Farmer	207	83.1
	Non-farmer	42	16.9
Mother’s profession	Farmer	232	93.2
	Non-farmer	17	6.8

Gomido et al. (35).

Table 1 shows that the number of schoolchildren who took part in the study is 249, 60.2% are boys and 39.8% are girls. Pupils at level 1 (CI-CE1) are more numerous than those at level 2 (CE2-CM2). The parents of these schoolchildren are mostly farmers with 83.1% of the fathers and 93.2% of the mothers.

### Socio-demographic characteristics of respondents in the municipality of Ze

Table 2 presents the socio-demographic characteristics of schoolchildren in the municipality of Ze. It shows that three hundred fifty-two (352) schoolchildren participated in the study. It is counted 56.53% of boys and 43.47% of girls. Level 2 pupils (CE2-CM2) are slightly more numerous than those at level 1 (CI-CE1) with a difference of 2.84%. The parents of these schoolchildren are mostly farmers, with 54.83% among the fathers and 47.44% among the mothers.

**TABLE 2:** Socio-demographic characteristics of Ze schoolchildren.

Variables	Modalities	Numbers (*n* = 352)	Frequency (%)
School	Sèdjè-Dénou/A	118	33.52
	Sèdjè-Dénou/B	94	26.70
	Agbohounsou	55	15.63
	Aguiakpa	85	24.15
Sex	Female	153	43.47
	Male	199	56.53
Age	(5–10 ans)	212	60.23
	(11–16 ans)	140	39.77
School level	Level 1 (CI-CE1)	171	48.58
	Level 2 (CE2-CM2)	181	51.42
Father’s profession	Farmer	193	54.83
	Non-farmer	130	36.93
	Other	29	8.23
Mother’s profession	Farmer	167	47.44
	Non-farmer	171	48.58
	Other	14	3.97

Field work, 2016.

### Baseline prevalence of soil-transmitted helminthiasis in the municipality of Lalo and Ze

**FIGURE 2:**
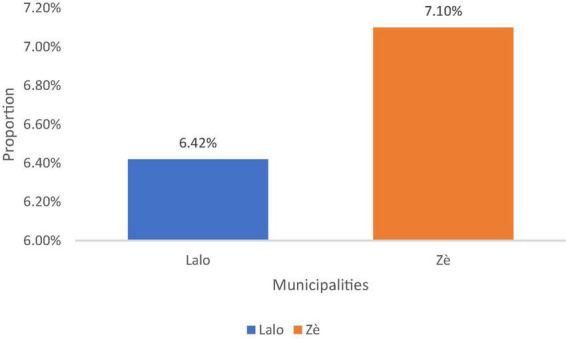
Prevalence of soil-transmitted helminths in Lalo and Ze. Gomido et al. (35); Field work, 2016.

Figure 2 presents the prevalence of soil-transmitted helminthiasis in both municipalities. The prevalence of soil-transmitted helminthiasis in the commune of Lalo was 6.42%. In the commune of Ze, the results related to the prevalence of soil-transmitted helminthiasis shows that the prevalence of soil-transmitted helminthiasis in the commune of Ze was 7.10%.

In municipality of Lalo, the helminth species identified are *Ascaris lumbricoides* (2.8%), *Trichuris trichiura* (2.41%), *Ankylostoma duodenale* (1.20%) as shown in Supplementary Figure 1. However, the helminth species identified in Ze are *Ascaris lumbricoides* (6.82%); *Trichuris trichiura* (0.28%). No case of *Ankylostoma duodenale* was detected (Supplementary Figure 1).

### Associated factors with soil-transmitted helminthiasis in municipality of Lalo

The results obtained from the bivariate analysis of the factors associated with the prevalence of soil-transmitted helminthiasis in the commune of Lalo are as follows (Table 3). From the analysis of Table 3, it appears that only hand washing before meals is statistically significant and therefore associated with the prevalence of soil-transmitted helminthiasis with an OR = 3.16 (95% CI = 1.19–8.34).

**TABLE 3:** Bivariate analysis of factors associated with soil-transmitted helminths in the commune of Lalo.

Variables	Modalities	No	Yes	OR (95% CI)	*p*
Sex (%)	Female	93 (93.9)	6 (6.1)	0.90 (0.34–2.42)	1.0*
	Male	140 (93.3)	10 (6.7)		
Age (year)	4–10	174 (95.1)	9 (4.9)	0.46 (0.18–1.19	0.14**
	11–15	59 (89.4)	7 (10.6)		
School level	CI–CE1	158 (95.2)	8 (4.8)	0.5 (0.19–1.28)	0.17*
	CE2–CM2	75 (90.4)	8 (9.6)		
Water supply source	Drilling/Tap water	225 (93.8)	15 (6.3)	0.56 (0.08–3.8)	0.46 **
	Surface water/rain	8 (88.9)	1 (11.1)		
Handwashing before meals	No	76 (88.4)	10 (11.6)	3.16 (1.19–8.34)	0.03*
	Yes	157 (96.3)	6 (3.7)		
Fruit washing	No	193 (92.8)	15 (7.2)	2.96 (0.40–21.77)	0.48**
	Yes	40 (97.6)	1 (2.4)		
Washing hands after toilet	No	144 (92.9)	11 (7.1)	1.33 (0.48–3.72)	0.61*
	Yes	89 (94.7)	5 (5.3)		
Father’s profession	Farmer	192 (92.8)	15 (7.2)	3.04 (0.41–22.41)	0.49**
	Non-farmer	41 (97.6)	1 (2.4)		
Mother’s profession	Farmer	218 (94.0)	14 (6.0)	0.5 (0.13–2.10)	0.3**
	Non-farmer	15 (88.2)	2 (11.2)		
Sources of water used	Water/rain	225 (93.8)	15 (6.3)	0.56 (0.08–3.80)	0.5**
	Surface water/rain	8 (88.9)	1 (11.1)		
Waste management	Incineration/landfilling	119 (93.0)	9 (7.0)	1.2 (0.47–3.16)	0.8*
	Dump/nature	114 (94.2)	7 (5.8)		

Gomido et al. (35). *Chi-square test. **Fischer test. OR, Odds Ratio. Mann-Whitney *U*-test. N/A, not applicable.

### Associated factors with soil-transmitted helminthiasis in municipality of Ze

The bivariate analysis of the factors associated with soil-transmitted helminthiasis in the commune of Ze is presented in Table 4.

**TABLE 4:** Bivariate analysis of factors associated with soil-transmitted helminths in the commune of Ze.

Variables	Modalities	Yes	No	OR	IC à 95%	*P*-value
Age (year)	5–10	13 (6.13)	199 (93.87)	1.43	(0.63–3.24)	*
	11–16	12 (8.57)	128 (91.43)			0.383
School level	Level 1	14 (8.19)	157 (91.81)	0.72	(0.31–1.64)	*
	Level 2	11 (6.08)	170 (93.92)			0.441
Sex	**Female**	**3 (1.96)**	**150 (98.04)**	**6.21**	(1.82–21.17)	**
	**Male**	**22 (11.06)**	**177 (88.94)**			**0.001**
Household water supply	Clean water	13 (6.22)	196 (93.78)	1.38	(0.61–3.12)	0.435*
	Non-clean water	12 (8.39)	131 (91.61)			
Drinking water at school	Clean water	21 (7.42)	263 (92.58)	0.76	(0.25–2.31)	**
	Non-clean water	4 (5.80)	64 (94.20)			0.796
Washing fruits before eating	Yes	2 (2.53)	77 (97.47)	3.5	(0.81–15.36)	**
	No	23 (8.42)	250 (91.58)			0.083
Handwashing before meals	Yes	16 (8.08)	182 (91.92)	0.70	(0.30–1.64)	*
	No	9 (5.84)	145 (94.16)			0.417
Handwashing with soap and water	Simple water	12 (7.14)	156 (92.86)	0.98	(0.43–2.23)	*
	Water + Soap	13 (7.07)	171 (92.93)			0.977
Washing the body every day	Yes	19 (6.17)	289 (93.83)	0.41	(0.15–1.10)	*
	No	6 (13.64)	38 (86.36)			0.071
Washing hands after toilet	Yes	8 (7.08)	105 (92.92)	0.99	(0.41–2.37)	*
	No	17 (7.11)	222 (92.89)			0.990
Wearing shoes always	Yes	10 (8.47)	108 (91.53)	0.73	(0.32–1.70)	*
	No	15 (6.41)	219 (93.59)			0.476
No use of toilets at school	Yes	23 (7.67)	278 (92.33)	2.07	(0.47–9.08)	**
	No	2 (3.85)	49 (96.15)			0.556
Availability of home latrine	Yes	7 (5.47)	121 (94.53)	0.66	(0.26–1.63)	*
	No	18 (8.04)	206 (91.96)			0.367
Father’s profession	Farmer	9 (6.98)	120 (93.02)	0.97	(0.41–2.26)	*
	Non-farmer	16 (7.17)	207 (92.83)			0.944
Mother’s profession	Farmer	5 (10.64)	42 (89.36)	1.69	(0.60–4.76)	*
	Non-farmer	20 (6.56)	285 (93.44)			0.310

Field work, 2016. OR, odds ratio; CI, confidence interval. *Chi-square test. **Fischer test. The bold value express the association between sex and STH morbidity.

It appears from Table 4 that the *P*-value of the Fisher test is 0.001. It is therefore concluded that the prevalence of soil-transmitted helminthiasis (3/153, i.e., a percentage of 1.96%) in the group of female schoolchildren is statistically lower than the prevalence of soil-transmitted helminthiasis (22/199, i.e., a percentage of 11.06%) in the group of male schoolchildren.

Considering socio-demographic characteristics, apart from sex which is associated with these morbidities, the age group 5 to 10 years has a lower prevalence (6.13%) than that of 11 to 16 years (8.57%); but this difference is not statistically significant.

With regard to personal hygiene, even if the washing of fruit is not statistically associated with the prevalence of soil-transmitted helminthiasis, it should be noted that only 2.53% of those who regularly wash fruit carry soil-transmitted helminths against 8.42% of those who said they never wash fruit. The prevalence of soil-transmitted helminthiasis in schoolchildren who do not wear shoes remains high (8.47%) compared to those who do (6.41%) even though this difference is not statistically significant. With regard to collective behavior, even if the availability of a latrine at home is not significantly associated with the intestinal worms studied, we note that the prevalence remains high (8.04%) in the group of those who do not have a toilet latrine compared to those who have one (5.47%).

### Description of the interventions implemented in the municipalities of Lalo and Ze

#### Raising awareness among schoolchildren using CHAST method

Schoolchildren were made aware of good hygiene and sanitation practices through CHAST method (15). The themes developed during these sessions relate to personal hygiene, hand washing, consumption of good quality water, waste management, the health consequences of the low level of WASH, the ways of transmission and prevention of intestinal parasites.

#### WASH infrastructures

Water, Hygiene and Sanitation infrastructures have been built in schools. These are improved water sources such as boreholes equipped with solar panels and human motor pumps, human powered drilling, hand-washing devices, ECOSAN (Ecologic Sanitation) type latrines and the donation of liquid soap such as illustrated by Photo 1. In order to make soap available and to facilitate its accessibility to schoolchildren, parents of students, especially mothers were trained in the preparation of liquid and solid soap (Supplementary Photo 1).

### Baseline informations of wound hygiene practices

Several practices that can be qualified as harmful in the management of wounds have been identified in the community. These harmful practices are; using scalding hot water on wounds, placing sand or ash on wounds to repel flies, using herbal medicines and applying powder from antibiotic capsules to dry wounds. However, some non-harmful practices have been noted such as the use of soap and plants such as *Ocimum gratissimum* (Table 5). Wound screening sessions were organized in the community. During these sessions, several types of wounds were diagnosed, most of which are traumatic wounds, chronic ulcers, leprosy plantar ulcers, Buruli ulcers and other types of wounds. Some wounds encountered are presented on Photo 2.

**TABLE 5:** Products commonly used to treat wounds at home classified according to the reasons for their use.

Practices and products commonly used	Reason for use
Hot water on the wound	Reduces pain and cleans the wound
	Remove pus from the wound
Cleaning soap	Cleans the wound
*Ocimum gratissimum*	Cleans the wound
Sand/ash	Protects against insects
Lemon	Stops the bleeding
Toothpaste	Dries hot wounds
Antibiotic capsules used externally	Dries wounds
Ibuprofen	Reduces pain
Powdered herb, bark or roots	Dries wounds
Black soap prepared with plants	Dries wounds
Agatouman (*Chromolaena odorata*)	Stops bleeding
Banana tree sap	Treatment
Hyssop (Hysopes officinalis)	Treatment
Red oil	Treatment
Shea butter	Scar management
Snail shell	Dries hot wounds
Petroleum and brake oil	Treatment

Field work, 2021.

### Description of wound hygiene practices

#### Community awareness on wound care

The communities were made aware of five themes related to wound care, which are: the importance of good wound care, healing indicators, danger signs of a wound, wound management and scar management.

#### Summary description of the participants in the awareness sessions

Summary description of the participants in the awareness sessions (Supplementary Table 1) presents the socio-professional categories and the villages of origin of the participants in the awareness sessions. From this table, we see that the participants were predominantly female, i.e., 25.8% female children and 24.2% female adults. Next, come male children (21.8%) and male adults (20.7%).

#### Screening and care of wounds

The screening of all cases of wounds systematically follows each awareness session. The care of the wounds was done individually with respect for the privacy of the patients by the well-trained nursing staff. During wound management, the basic notions of wound hygiene are taught to patients and their families. The patient and the family are then invited to describe the different stages of wound hygiene that have been taught to them so that the team can ensure that the concepts taught are well-understood (Photo 3).

Each patient receives a kit containing a bar of soap, shea butter, bandages for home-based wound hygiene. Pain medication is given if needed (Photo 4). Finally, the patient is entrusted to a member of a support group close to his locality for his follow-up in the application of the knowledge acquired during the awareness session.

One hundred and fifty-eight (158) cases of wounds were detected in the 08 villages, namely: Tan, Don Aliho, Don Tohomè de (District of Don-Tan), Doga Alicon (District of Zagnanado center), Klobo, Dizigo, Sèdjè Dénou (District of Dovi-Dovè), and Bamè (District of Agonlin Houègbo). Supplementary Table 2 presents the socio-demographic characteristics of the carriers of the detected lesions. We note from this table that, the majority of carriers of lesions (wounds) are male (61.4%). Most of the cases detected are married (59.5%). Schoolchildren represent the predominant occupational group (38.0%) followed by housewives (16.5%). The lesions detected are shown in Supplementary Figure 2. That Figure shows that the lesions screened for are mostly wounds (open lesions).

### Post-interventions assessment

#### In the municipality of Lalo

**FIGURE 3:**
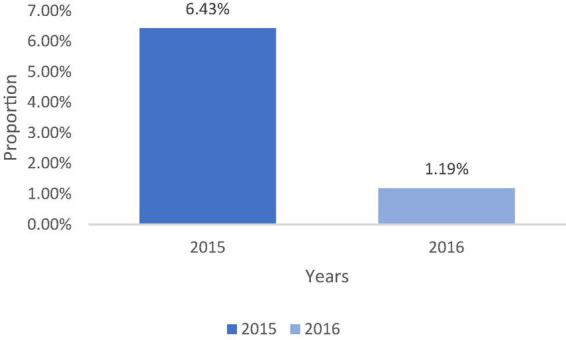
Evolution of the prevalence of soil-transmitted helminthiasis between 2015 and 2016 in Lalo. Gomido et al. (35).

The prevalence of soil-transmitted helminthiasis is presented at the end of the evaluation. It has been noted the decrease of soil-transmitted helminthiasis prevalence from 6.52 to 1.19% (Figure 3).

#### In the municipality of Ze

**FIGURE 4:**
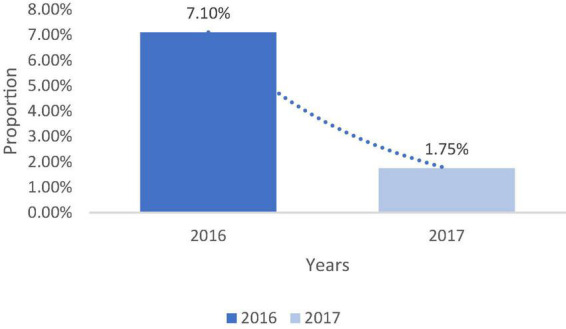
Evolution of the prevalence of soil-transmitted helminthiasis between 2016 and 2017 in Zè. Field work, 2017.

The new prevalence of soil-transmitted helminthiasis in the commune of Ze is 1.75%. It appears that the prevalence of soil-transmitted helminthiasis, which was 7.10%, decrease to 1.75% after interventions (Figure 4).

### In the municipality of Zagnanado

Follow-up of screened wounds have been organized. Of the 83 wound cases, 61 or 73.5% were healed and 13 (15.7%) were healing. This testifies to the effectiveness of the treatment if it is well applied. Below some images taken during the follow-up (Photo 5).

## Discussion

The objective of the present study was to evaluate the effect of WASH on soil-transmitted helminth infections, and wound hygiene. It addresses the issue of the links between water, sanitation and human health in vulnerable populations. It took place in three municipalities in Benin and was conducted by a multidisciplinary team made up of WASH sector specialists, sociologists, epidemiologists, and nurses specializing in the treatment of skin diseases. It took place in five phases.

In the first phase of the study, a prevalence of soil-transmitted helminthiasis was 6.42% in the commune of Lalo and 7.10% in the commune of Ze. Similar studies had been carried out by other authors (19–21). A comparison of the results shows that the prevalences obtained by the present study are low compared to that of 12.12% obtained by Ibikounlé et al. In the commune of Sô-Ava in southern Benin in 2013 (21). They are also lower than those of 43.3% of Echazu and al. in Argentina (22) et 10.7% from Erismann and al. in Burkina Faso (23).

The Kato-Katz technique used is recommended by the World Health Organization. However, there are several other methods such as direct microscopy, formalin-ether concentration (FEC), McMaster, FLOTAC, and Mini-FLOTAC.

The identification tests of the helminth species allowed to obtain for the species *Ascaris lumbricoides* a prevalence of 2.8% in Lalo and in Zè, a prevalence of 6, 82%. Which is different from the results obtained by Mohaghegh et al. in Iran where no eggs of ascaris were found (24).

However, these prevalences of *Ascaris lumbricoides* are higher than those obtained in Morocco by Eiqaj et al., where the prevalence of this species is 1.23% (25). These prevalences of *Ascaris lumbricoides* are also much lower than the 17.8% obtained by Dankoni and Tchuenté in Cameroon (26).

For the *Trichuris trichiura* species, the prevalence obtained in Lalo is 2.41% and that obtained in Zè is 0.28%. These values are lower than the 3.12% obtained by Mohaghegh et al. (24) and the 3.07% obtained by Eiqaj et al. in 2009 in Morocco (25).

In Lalo a prevalence of 1.20% for Ankylostoma duodenale was obtained and no cases of this species were detected in Zè. The prevalence for this geohelminth species is thus lower than the 2.1% obtained by Mohaghegh et al. in Iran (24).

Interventions based on the promotion of hygiene and sanitation among schools and school children have reduced the prevalence of soil-transmitted helminth infections from 6.42 to 1.19% in the commune of Lalo and from 7.10 to 1.75% in the municipality of Ze. This decrease in geohelminthiasis prevalence between the baseline and post-intervention phases could be explained by the actions implemented, notably the provision of WASH infrastructure, education on good hygiene and sanitation practices and deworming.

The results of the study are similar to those obtained by Gizaw and al. in Ethiopia (27). These results are also comparable to those obtained by Mado and al. in Tanzania (28). Another study carried out in the commune of Bonou in Benin demonstrated a reduction in the incidence of Buruli ulcer in populations with wells, compared to those without (29).

Similar results were obtained in Kenya in trachoma control. Indeed, the authors have shown that regular washing of the face and hands with clean water associated with education improves knowledge about trachoma and reduces the risk of contracting this disease (30). The work done by Waite et al. reveals progress in collaboration between the WASH and NTD sectors, leading to progress in several programming areas; research; advocacy and policy; training and capacity building; mapping, data collection and monitoring (31).

Our study also looked at the effects of WASH interventions on NTDs with skin manifestations. Awareness sessions on good wound hygiene practices (use of drinking water, washing of hands and wounds with soap, sanitation of the living environment) for good and rapid healing of wounds were conducted. These awareness sessions were followed by the donation of a dressing kit containing soap. It was noticed at the end of the interventions a healing of the wounds in people carrying wounds for several years which did not heal. Similar results showing the effect of WASH interventions on NTDs in general and NTDs with skin manifestations in particular had been observed by several authors. The study by Emerson and al. revealed that WASH factors including water source, facility access to water, access to soap, hand washing practices and open defection are linked to leprosy infection (32). Also, Savage and al. concluded in their study that WASH interventions are a necessity to prevent continued transmission and reinfection with NTDs such as trachoma and intestinal worms (33). According to them, unless WASH problems are properly addressed, NTDs will not be eliminated in the long term. Also Johnson and al, found in their study in Benin that the type of housing as an indicator of the socioeconomic status, the permanent availability of soap and improved hygiene practices were identified as the main factors positively associated with improved sanitation status The type of housing as an indicator of the socioeconomic status, the permanent availability of soap and improved hygiene practices were identified as the main factors positively associated with improved sanitation status (34).

## Conclusion

The municipalities of Lalo, Ze, and Zagnanado, all rural municipality located in southern Benin, are endemic to several NTDs including soil-transmitted helminthiasis and NTDs with cutaneous manifestations. This formative research has made it possible to reduce the prevalence of soil-transmitted helminthiasis but also to contribute to the healing of several cases of wounds. It can therefore be concluded that WASH interventions are necessary in the NTDs control.

## Data availability statement

The original contributions presented in this study are included in the article/Supplementary material, further inquiries can be directed to the corresponding authors.

## Ethics statement

The studies involving human participants were reviewed and approved by the Ethics Committee for Health Research of Benin (No. 123/MS/DC/SGM/ + DFR/CNERS/SA, and No. 43 of November 23, 2017, No. 5/MS/DC/SGM/DRFMT/CNERS/SA). Written informed consent to participate in this study was provided by the participants’ legal guardian/next of kin.

## Author contributions

RJ oversaw the entire process of implementing the intervention. All authors actively participated in the development of the research proposal, its implementation, the statistical and laboratory analysis and the interpretation of the results, charge of writing various parts of the research report, read and made comments on the manuscript, and which have been taken into account in the final version of the manuscript.
