# Supplementary material for: WASH and NTDs: Outcomes and lessons learned from the implementation of a formative research study in NTD skin co-endemic communities in Benin
Source: Front Med (Lausanne). 2023 Feb 28;10:1022314. doi: 10.3389/fmed.2023.1022314 (PMC10011489; doi:10.3389/fmed.2023.1022314)
Supplement: Supplementary file 1 [file Data_Sheet_1.pdf]

Supplementary Material

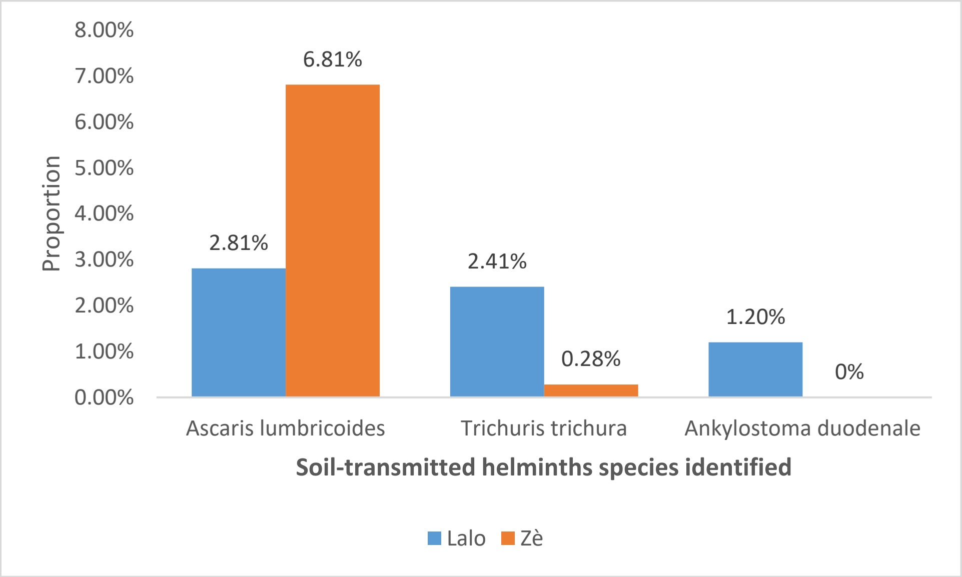

Figure S1: Soil-transmitted helminths species identified in Lalo and Ze

Sources : Gomido et *al.* 2019 ; Field work, 201

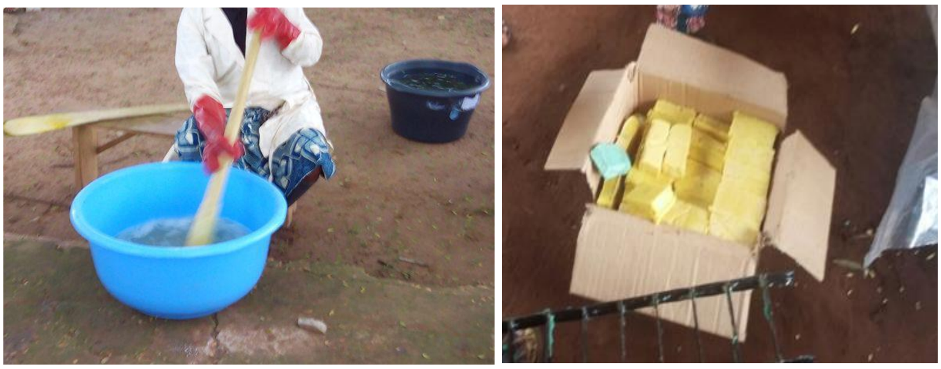

Photo S1: Preparation of liquid soap and prepared solid soaps
Source : field work, 2017

Table S1: Summary description of participants in the outreach sessions

Villages	Female children 2 to 17 years old	Male children 2 to 17 years old	Female adults 18 years and older	Adult males 18 years and older	Teachers	Opinion leaders	Religious leaders	Crowned heads	Health workers	Local authorities	Various groups	Total number
Tan	43	36	41	32	2	5	1	0	1	3	2	**164**
Don Aliho	33	29	31	39	3	4	0	0	1	2	1	**142**
Don Tohomè	31	35	36	31	1	2	2	0	1	1	1	**140**
Doga Alicon	38	32	29	25	1	3	2	0	1	3	2	**134**
Dovi Klobo	35	26	32	27	0	3	1	0	1	2	1	**127**
Dovi Dizigo	40	31	38	29	3	5	3	1	1	2	2	**153**
Dovi Zounnou	38	30	34	25	0	3	2	0	1	3	1	**136**
Bamè	41	34	39	32	3	6	3	0	1	3	2	**162**
**Total**	**299 (25,8%)**	**253 (21,8%)**	**280 (24,2%)**	**240 (20,7%)**	**13 (1,1%)**	**31 (2,7%)**	**14 (1,2%)**	**1 (0,1%)**	**8 (0,7%)**	**19 (1,6%)**	**12 (1,0%)**	**1158 (100,0%)**

Table S2: Socio-demographic characteristics of screened lesion carriers

Characteristic	Number	Percentage (%)
**Sex**		
Female	61	38,6
Male	97	61,4
**Total**	**158**	**100,0**
**Marital status**		
Single	57	36,1
Married	94	59,5
Widower	7	4,4
**Total**	**158**	**100,0**
**Profession**		
Income-generating activity	6	3,8
Apprentice	10	6,3
Motorcycle Taxi Driver	2	1,3
Tailor	4	2,6
Farmer	22	13,9
Schoolchildren	60	38,0
Scholar	9	5,7
Student	3	1,9
Mechanic	1	0,6
Housekeeper	26	16,5
Carpenter	3	1,9
None	9	5,7
Painter	1	0,6
Relay	1	0,6
Weaver	1	0,6
**Total**	**158**	**100,0**

Source *: Field work, February-March 2021*

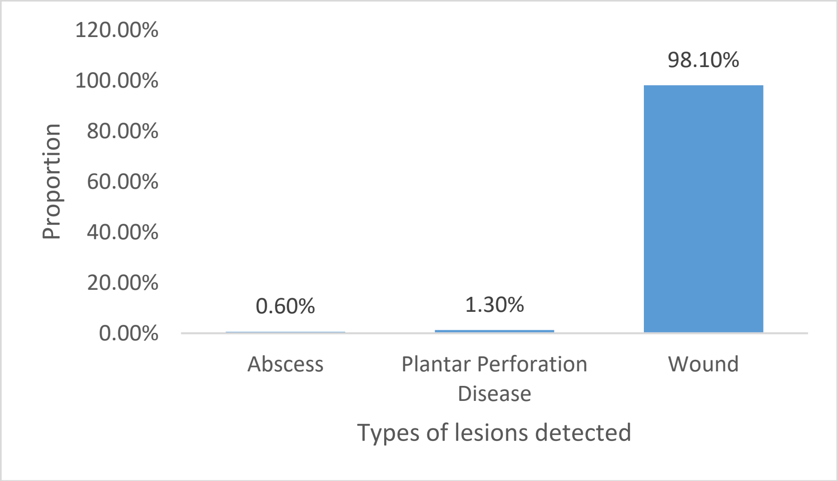

Figure S2: Types of lesions detected during sensitizations

Source *: Field work, February-March 2021*
